# Managing rare fractures – a case report of an isolated avulsion fracture of the peroneus longus tendon

**DOI:** 10.1093/jscr/rjac311

**Published:** 2022-07-05

**Authors:** Raghav Nand, Muhammad Murtaza Khan, Dakshinamurthy Sunderamoorthy

**Affiliations:** Trauma and Orthopedics, Scunthorpe General Hospital, Scunthorpe, North Lincolnshire and Goole Hospital Trust, UK; Trauma and Orthopedics, Scunthorpe General Hospital, Scunthorpe, North Lincolnshire and Goole Hospital Trust, UK; Trauma and Orthopedics, Scunthorpe General Hospital, Scunthorpe, North Lincolnshire and Goole Hospital Trust, UK

## Abstract

Avulsion fractures of the peroneus longus tendon are seldom seen and potentially can go undiagnosed during an emergency visit. If not managed appropriately, it can lead to chronic pain and suffering. This case report presents a 55-year-old postman who was seen in the clinic complaining of persistent pain over the instep of his right foot with no history of trauma. His pain was localized to the first metatarsophalangeal joint with some radiation to the heel. Magnetic resonance imaging revealed an isolated avulsion fracture of the first metatarsal, which was initially missed on X-ray. In this case, the patient was successfully treated with a mixture of steroid and local anesthetic injections. Following our intervention, the Manchester Oxford Foot Questionnaire was reduced from 33 to 0. The goal of this article is to raise awareness of this rare finding for doctors who may face this in accident and emergency (A&E), Orthopedic clinics or at a general practice (GP) practice.

## INTRODUCTION

An isolated fracture of the peroneus longus tendon at the base of the first metatarsal joint is a rare entity often unable to diagnose using X-ray upon the initial presentation. There are no gold standard treatments by which these injuries can be managed. Various case reports have shown where surgeons have used a combination of their own personal preference and the clinical picture in front of them. Management options vary from conservative using a lower leg cast [1] and restricted physical exertion with regular follow-up [2]. In the case of failed conservative management, we must also consider surgery, which includes percutaneous fixation [3] or excision of the non-healed fracture fragment and arthrodesis [1]. This case therefore possesses both a diagnostic and management challenge to doctors who face it.

## CASE REPORT

This case report brings to light a 55-year-old postman who presented to clinic with pain in his right foot for over 2 months. The pain was confined to the right first metatarsophalangeal joint with occasional radiation to instep and over the heel. This pain was in association with occasional cramping in his right calf muscle and was aggravated by standing or walking and relieved at rest. There was no history of trauma to his foot and this pain was insidious in onset.

Upon examination, he was able to stand erect with plantigrade feet. There was a prominent bunion over the right foot. Both heels were in normal valgus alignment and went into Varus on standing at the tip of toes.

Further examination revealed, the patient was tender over the posteromedial aspect of the calf just above the heel and at the knot of Henry. It was noted that the pain got worse on hyperextension at the first metatarsophalangeal joint. The patient also had hallux valgus of moderate degree that was partially correctable. The affected foot itself demonstrated a satisfactory range of motion at the ankle, subtalar, mid foot and forefoot. The distal neurovascular status was found to be unremarkable.

**Figure 1:**
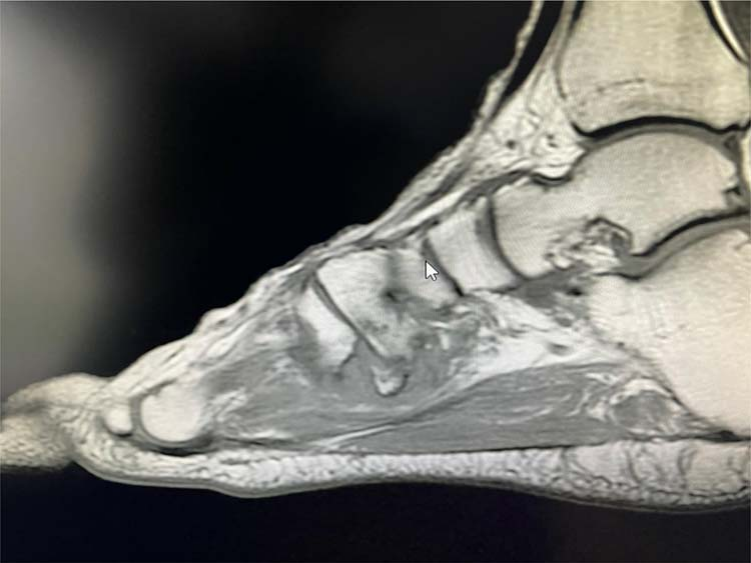
T1 sagittal view showing non-union of the avulsion fracture.

**Figure 2:**
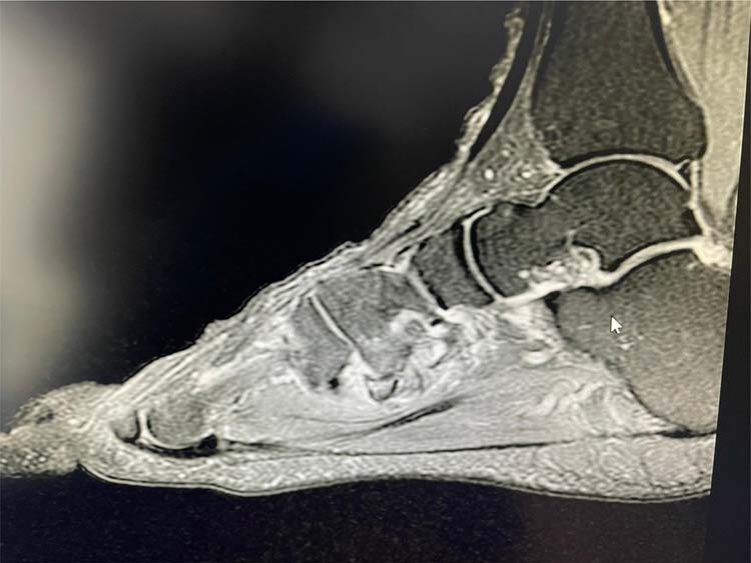
T2 sagittal view showing non-union of the avulsion fracture.

**Figure 3:**
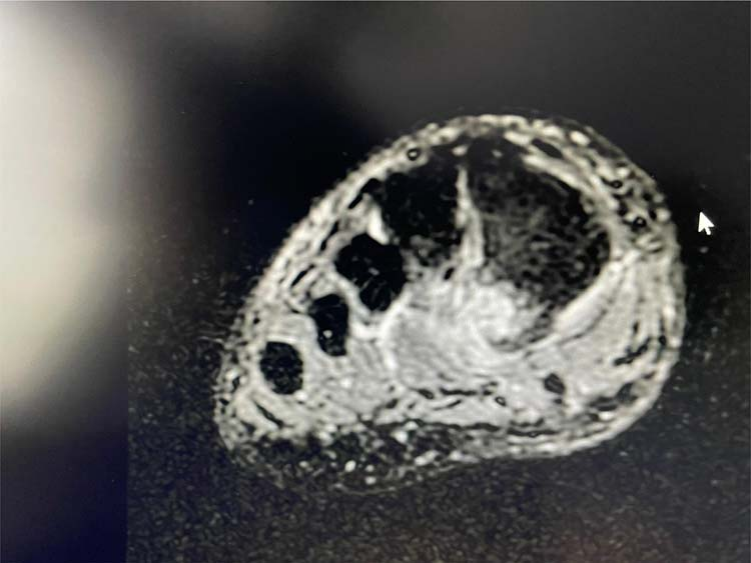
Coronal T2 view showing the non-union of the first metatarsal avulsion fracture.

Based on this examination, the initial impression was tendinosis of the flexor hallucis longus. To confirm this diagnosis and rule out any other bony injury, a magnetic resonance imaging scan was requested of the right foot and ankle. Imaging revealed an avulsion of peroneus longus tendon at base of first metatarsal with a possible non-union as well (Figs 1–3).

This case was further discussed in the regional foot and ankle meeting. The outcome was to inject the painful nonunion located at the base of the first metatarsal on the plantar surface of the foot with a mixture of local anesthetic and steroid injections. Post procedure there remained no neurovascular deficit. The patient was reviewed at 3 months and his pain score and functional outcome improved significantly. Moreover, following our intervention, the Manchester Oxford Foot Questionnaire reduced from 33 to 0. At the 1-year follow-up, he remained symptom-free and discharged.

## DISCUSSION

The peroneus longus tendon has a role in eversion and planter flexion of foot along with providing stabilization to arches of foot [1]. The pattern of injury to this tendon is based on two factors: one is the mechanism of insult, if injured, and second is the variation in the insertion pattern of peroneus longus tendon itself. The common agreed mechanism of injury is forced dorsiflexion and inversion [2]. Moreover, the shear tensile force on the peroneus longus tendon is also considered as a contributory factor to injury [4]. Recent cadaveric dissection has also recognised a variation in tendon insertion which includes insertion at the lateral aspect of cuboid and lateral aspect of calcaneum, fifth metatarsal bone and fibular trochlea of calcaneum [5].

Although both operative and conservative methods of treatment have been documented in the literature with a variability of success rates, the choice varies on the type of injury, treating surgeon and most importantly the patient. There are some notable factors which do favor non-operative treatment compared to surgery. These involve the size and displacement of fracture. Studies show that small fragments or minimally displaced, undisplaced or multi-fragmental fracture patterns will do better with non-operative treatment [2].

Hodor *et al*. describes a non-weight bearing strategy of conservative treatment as a choice of regimen for this type of injury; however, Zermatten *et al*.’s findings were not supportive of this therapy as they were of the view that patient will represent with a painful non-union at 6 months and will require excision of the bony fragment along with arthrodesis following a trial conservative treatment [3].

Literature regarding the role of physiotherapy in relation to these injuries is limited, and at the moment, there are no specific guidelines in practice. Some researchers have claimed to treat chronic ankle pain in patients following multiple subtle injuries to their joint by means of physiotherapy to provide an improved functional ability [6].

## CONCLUSION

In summary, isolated avulsion fractures at the site of insertion of the peroneus longus tendon are rare injuries. It is important to be aware of such injuries when patients present with pain over the instep. In this case a combination of local anesthetic mixed with steroids was a successful way of managing an ununited avulsion fracture however it is imperative to always take patient selection and clinical judgement into account in managing such injuries.
